# From Molecules to Life: Quantifying the Complexity of Chemical and Biological Systems in the Universe

**DOI:** 10.1007/s00239-017-9824-6

**Published:** 2017-12-19

**Authors:** Thomas Böttcher

**Affiliations:** Department of Chemistry, Konstanz Research School Chemical Biology, Zukunftskolleg, University of Konstanz, Konstanz, Germany

**Keywords:** Life-like system, Evolution, Biomolecule, Chemical complexity, Biogenic unit

## Abstract

Life is a complex phenomenon and much research has been devoted to both understanding its origins from prebiotic chemistry and discovering life beyond Earth. Yet, it has remained elusive how to quantify this complexity and how to compare chemical and biological units on one common scale. Here, a mathematical description of molecular complexity was applied allowing to quantitatively assess complexity of chemical structures. This in combination with the orthogonal measure of information complexity resulted in a two-dimensional complexity space ranging over the entire spectrum from molecules to organisms. Entities with a certain level of information complexity directly require a functionally complex mechanism for their production or replication and are hence indicative for life-like systems. In order to describe entities combining molecular and information complexity, the term biogenic unit was introduced. Exemplified biogenic unit complexities were calculated for ribozymes, protein enzymes, multimeric protein complexes, and even an entire virus particle. Complexities of prokaryotic and eukaryotic cells, as well as multicellular organisms, were estimated. Thereby distinct evolutionary stages in complexity space were identified. The here developed approach to compare the complexity of biogenic units allows for the first time to address the gradual characteristics of prebiotic and life-like systems without the need for a definition of life. This operational concept may guide our search for life in the Universe, and it may direct the investigations of prebiotic trajectories that lead towards the evolution of complexity at the origins of life.

**Electronic supplementary material:**

The online version of this article (10.1007/s00239-017-9824-6) contains supplementary material, which is available to authorized users.

## Introduction

In past decades, planetary science has identified multiple places in the solar system such as Jupiter moon Europa and Saturn moon Enceladus that indicate active geology and presence of liquid water (Soderlund et al. 2014; Waite et al. 2017)—both presumably prerequisites for the evolution of complex chemistry and the existence of life. Yet, it has remained elusive how to actually detect and identify life or interpret the complexity of possibly encountered prebiotic molecules. It has been argued that there could be forms of life so fundamentally different from life as we know it that it would be difficult to recognize them (Cleland 2005). Any kind of life or life-like systems, however, are dissipative by nature and will require complexity to part from an equilibrium state (Capra 2007). Thus, complexity may be a reliable marker for life-like processes that forms the natural basis for maintenance of structure, replication, mutation, and selection (Emmeche 1997). I will here present a conceptual framework for quantifying the complexity of chemical systems and demonstrate that in combination with orthogonal measures it also allows assessing complexity of life-like systems in the Universe.

Biological units are characterized by the complex chemistry that constitutes and defines their structural and functional integrity. Proteins, nucleic acids, polysaccharides, and many smaller compounds such as lipids, cofactors, vitamins, and hormones are important biomarkers indicative for life. Also most strategies for the search for life in the Universe aim to detect complex chemical compounds as indicators for life (Sephton and Botta 2008; Summons et al. 2008). In contrast somewhat less complex molecules may be representative for prebiotic processes (Pross 2005). So far, we are lacking a generally accepted definition of life and some authors have been pessimistic if such a definition is at all possible (Cleland and Chyba 2002; Luisi 1998; Oliver and Perry 2006). I will argue here that regardless of the definition of life or whether or not such a definition is possible at all, chemical complexity is a necessary prerequisite for any form of life or life-like system, i.e., whatever is defined as major characteristics of life, such as a genetic program, compartmentalization, metabolism, the capability to regenerate and adapt, or the ability to evolve—neither of them is possible without chemical complexity (Koshland 2002; Luisi 1998).

Vice versa, if we were to detect any system outside of Earth that involves the maintenance and perpetuation of complex chemistry it would be of immediate interest to the question of the origins of life on Earth and the search for extraterrestrial life in the Universe (Benner 2010). I will here for the first time apply the concept of chemical complexity to large biomolecules and demonstrate that this approach in combination with the orthogonal measure of information complexity can generate a universal complexity scale that allows correlating and quantitatively comparing prebiotic and life-like systems in the Universe.

## Quantifying Complexity

Numerous previous authors have recognized complexity as an aspect central to life (Emmeche 1997; Ruiz-Mirazo and Moreno 2012; Ruiz-Mirazo et al. 2004). One of the major quests of astrobiology could thus be re-formulated as the search for complex chemical systems in the Universe. Yet, we were lacking a universal quantitative concept allowing to assess complexity over the entire range from simple molecules to biological units. I will here use the term chemical complexity to quantify complexity of molecular structures which in contrast to other types of complexity (e.g., molecular interactions) is relatively simple and straight forward to quantify. Although various measures of chemical complexity have been introduced, each of them suffered from certain limitations which made them unsuitable for larger biomolecules (Barone and Chanon 2001; Bertz 1981; Böttcher 2016; Whitlock 1998). Most of these complexity indices were based on graph theory. As recently demonstrated, graph theoretical approaches lead to non-linear behaviors that contradict the additive principle of information theory and prevent simple modular calculations of biopolymers and their multimeric complexes (Böttcher 2016). Other indices, however, were rather insensitive to important structural parameters like skeletal structure, branching, chiral centers, and symmetry. To address these shortcomings, I recently developed an additive definition of molecular complexity which relies on information theory and abstracts the information content of a molecule from the degrees of freedom of its atom-based microenvironments (Böttcher 2016). The resulting index for molecular complexity *C*_m_ (Eq. 1) can be manually calculated for any given chemical structure as has been described in detail before.

1 $${C_{\text{m}}}=\mathop \sum \limits_{i} {d_i}~{e_i}~{s_i}~{\log _2}\left( {{V_i}~{b_i}} \right) - \frac{1}{2}\mathop \sum \limits_{j} {d_j}~{e_j}~{s_j}~{\log _2}\left( {{V_j}~{b_j}} \right).$$

**Fig. 1:**
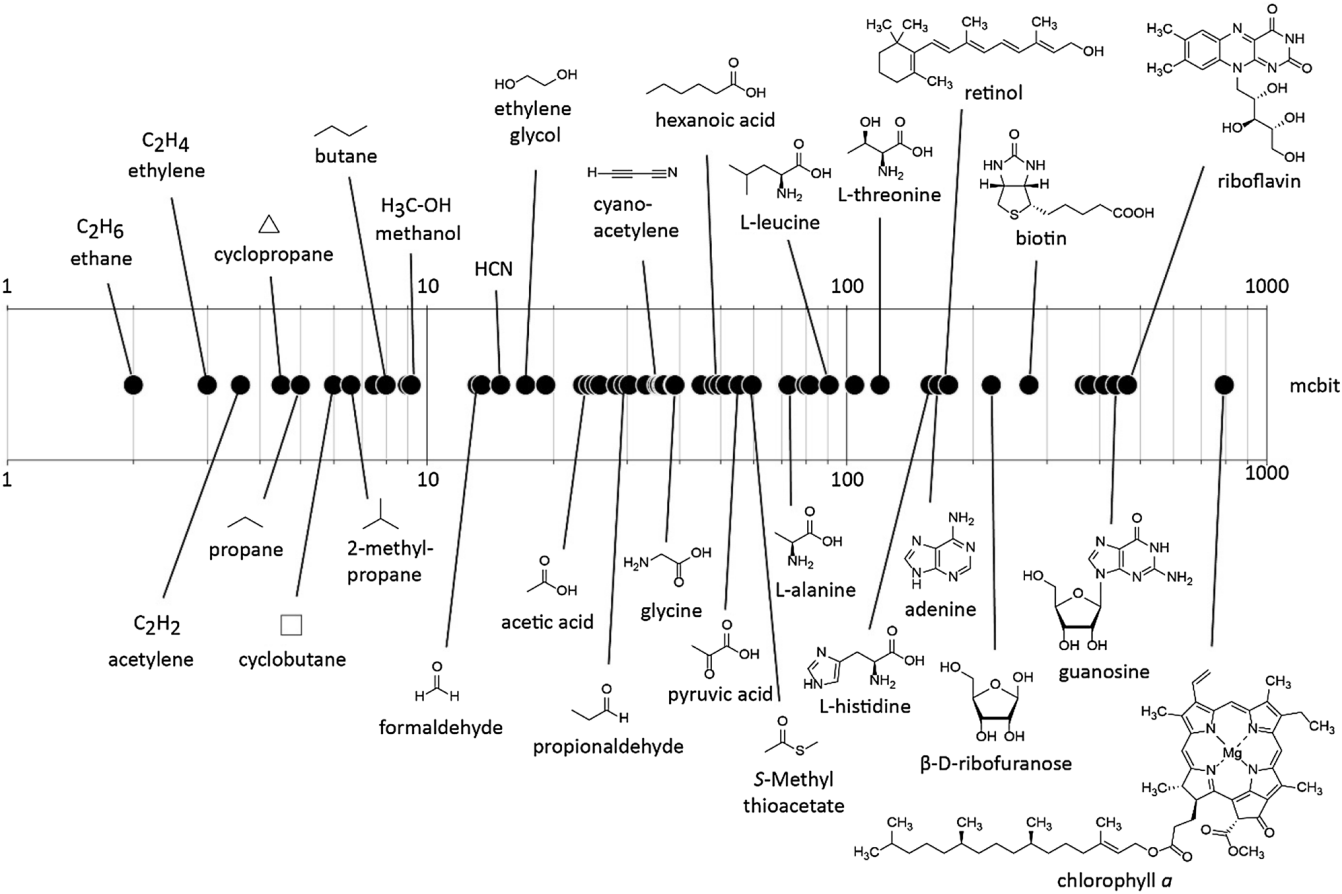
Molecular complexity *C*_m_ calculated for the structures of various small molecules in a universal complexity scale. The graphic representation is given in a logarithmic scale

In short, variables describing the microenvironments for every atom position *i* in a molecule are determined: the number of valence electrons *V*_*i*_ of the element of an atom, the total number of bonds *b*_*i*_ and the number of chemically non-equivalent bonds *d*_*i*_ to neighboring atoms with ${V_i}{b_i}>1$ (non-hydrogen atoms), the heteroatom diversity parameter *e*_*i*_, and the number of isomeric possibilities *s*_*i*_ at the *i*th position. Finally, symmetry is corrected for the corresponding *j*th symmetrical atom position of chemically equivalent sets of atoms. Using Eq. (1) allows to universally calculate the molecular complexity *C*_m_ of any chemical structure in bit of molecular complexity (mcbit). This method is particularly well suited in the whole range from small organic molecules to macromolecular complexes and non-covalent assemblages and will be applied here for the first time to biomolecules like proteins and nucleic acids (Böttcher 2016). However, chemical complexity can be calculated even for “exotic” molecules that are not based on carbon chemistry, such as boron or silicon-based compounds (Benner et al. 2004; Schulze-Makuch and Irwin 2006). It should be hereby noted that *C*_m_ is an intrinsic measure of molecular complexity and is not dependent on external conditions such as synthetic accessibility. As such, it does not change with technological progress or general advances in our knowledge about chemical reactions. Therefore, *C*_m_ is a universal measure and can be quantified for any chemical compound throughout space and time. Consequently, molecular complexity of entirely different molecular species can be compared on a universal scale of molecular complexity (Fig. 1).

This scale demonstrates, that the calculated molecular complexities roughly correlate with the rather arbitrary distinction of abiotic, prebiotic, and biotic molecules. For example, ethylene would not be considered as reliable biomarker as there are many abiotic processes that may lead to its formation. Amino acids, fatty acids, and simple sugars are typically considered as important prebiotic molecules, while chlorophyll *a* would be seen as strong indicator of biological activity. The concept of molecular complexity allows for the first time to quantitatively correlate and compare different compound classes on one universal scale. Classifying a molecule by its molecular complexity may be thus of great operational value for astrobiology. However, there are important limitations to using molecular complexity as the only index for measuring systems complexity of potential biomolecules. While molecular complexity is well suited to quantitatively compare the overall complexity of any chemical structure, it is not able to reflect the information content of a polymeric compound. A homopolymer like polyglycine may have the same molecular complexity as, for example, a functional protein enzyme, yet only the latter comprises sequence information. Large *C*_m_ values are thus necessary but not sufficient to indicate the presence of life-like systems. Therefore it may be useful to introduce orthogonal measures of complexity going beyond the molecular structure. Various types of complexity for biological systems have been distinguished ranging from compositional to behavioral complexity (Carbone and Narbonne 2014; Lynch and Conery 2003; Whitesides and Ismagilov 1999). Szostak and coworkers have developed the concept of functional information as measure for systems complexity (Hazen et al. 2007; Szostak 2003). While this approach is intriguing from a theoretical perspective, it is of only little practical applicability as it requires a detailed functional understanding of the components of a system, which we currently have not even achieved for the simplest unicellular organism and testing of maybe more than millions of structures in order to obtain the total or statistically representative fraction of configurations (e.g., RNA or protein sequences) which exhibit at least a specified degree of function (≥ *E*_*x*_). Furthermore, it remains unsolved how to integrate thousands to millions of distinct functions comprised in every cell of an organism as well as emergent functions that cannot be attributed to any discrete configuration of the system. Other concepts like the complexity of molecular interactions, spatial configuration, or behavior may be even more difficult to assess and currently lack an appropriate theoretical framework for their quantification. Consequently, the complexity of sequence information is the most applicable orthogonal measure which can be easily calculated for any molecule composed of a modular structure. The information content of a sequence of modules or building blocks can be deduced from information theory according to Shannon (Shannon 1948). In order to obtain an intrinsic measure of sequence information that is independent of the number and availability of putative building blocks in the native environment, calculations need to be based on their actual abundances in a given molecule. This step also avoids making vague assumptions on putative substrate preferences and mechanisms in prebiotic or extraterrestrial settings. Thus, sequence information complexity *C*_*i*_ is calculated directly from a sequence by equation (Eq. 2) where *p*_*k*_ is the probability of a chemical building block *k* (e.g., amino acid or nucleotide) being incorporated and *n*_*k*_ the number this building block occurs in a sequence.

2 $${C_i}= - \mathop \sum \limits_{k} {n_k}~{\log _{2~}}{p_k}.$$

The probability of incorporating each building block corresponds hereby to its relative frequency in the sequence. With *n* being the total number of building blocks in a molecule, the equation can formulated as follows:

3 $${C_i}= - \mathop \sum \limits_{k} {n_k}~{\log _{2~}}\frac{{{n_k}}}{n}.$$

A building block *k* describes the largest non-repetitive molecular frame, for instance, an amino acid or nucleotide. Accordingly, sequences of periodically alternating building blocks, such as certain polysaccharides, compounds with a regular crystal lattice, and homopolymers like cellulose or synthetic polyamides contain no sequence information (Figure S1). Also chemical and physical processes may discriminate the incorporation of certain building blocks into a sequence. Hereby the most conservative approach is taken by Eq. (3) for which knowing the sequence of a single macromolecule is sufficient to calculate its information complexity. Sequence information complexity is additive and can thus be applied also to non-covalent assemblies of multiple sequences. To distinguish molecular complexity values from sequence information complexity, I will here use the unit icbit for bit of information complexity.

The implications of the presence of a population of molecules or systems with sequence information are reaching beyond the actual information content as they necessitate an underlying mechanism that directs the synthesis of the sequence. The higher the information content of a sequence is, the more likely does it require an elaborate production or replication mechanism. Smaller peptides can be produced by non-ribosomal peptide synthases, molecular assembly lines consisting of multienzyme complexes, each consisting of multiple catalytic domains (Marahiel et al. 1997). The largest known non-ribosomal peptides, the polytheonamides consist of sequences of 48 amino acids (Hamada et al. 2005; Inoue et al. 2010). Proteins and nucleic acids with larger sequence lengths ranging from many hundred to millions of modular building blocks are generated in all known organisms by template-directed synthesis, either directly replicating the original molecule (e.g., DNA) or transcribing or translating its template sequence into a derived sequence (e.g., RNA and protein). However, different mechanisms may have been at work for the production of functional biogenic units in the early stages of evolution. One possible mechanism is cross-replication of units, which has been demonstrated to enable the self-sustained replication and evolution of ribozymes (Lincoln and Joyce 2009).

The probability of obtaining a population of molecules with identical sequences by combinatorial events without involving any elaborate production mechanism rapidly decreases with increasing sequence length so that already short sequences are highly unlikely to accumulate to any reasonable numbers by chance (Figure S2). In order to achieve the accurate production or replication of complex sequences, non-templated assembly lines as well as template-directed synthesis both require processes operating with highest fidelity. This involves selectivity in the recognition and discrimination of distinct building blocks and specificity regarding the chemical reactions carried out on them. Appropriately selective and specific processes are only possible with high-performance catalysts capable of molecular recognition and hence require functional structures comparable to known biocatalysts like protein enzymes or ribozymes. It can be thus concluded that complexity of sequence information goes hand in hand with the existence of a functionally complex mechanism for its production, irrespectively whether this involves template-directed or any kind of non-templated synthesis.

The degree of complexity may be indicative for the biogenicity of molecules and the presence of life-like systems. While any molecule with sequence information necessarily also exhibits molecular complexity, assemblies of larger units such as cells or organelles also contain components such as homopolymers, lipids, and metabolites that do not contribute sequence information but comprise molecular complexity (Figure S1). These units may be defined by their physicochemical barrier structures (e.g., membranes or capsules) or the interactions of their components (e.g., multicellular organisms or social insects). I will here introduce the term biogenic unit to classify systems combining molecular complexity and sequence information. Smaller biogenic units may be nested in larger ones reflecting the entire continuous spectrum from prebiotic units to life. Consequently, biogenic units can be individual molecules, multimeric complexes, single cells, organisms, or even societies. Any biogenic unit can now be described by a combination of molecular and sequence information complexity and quantified by a value pair (*C*_m_; *C*_*i*_).

**Fig. 2:**
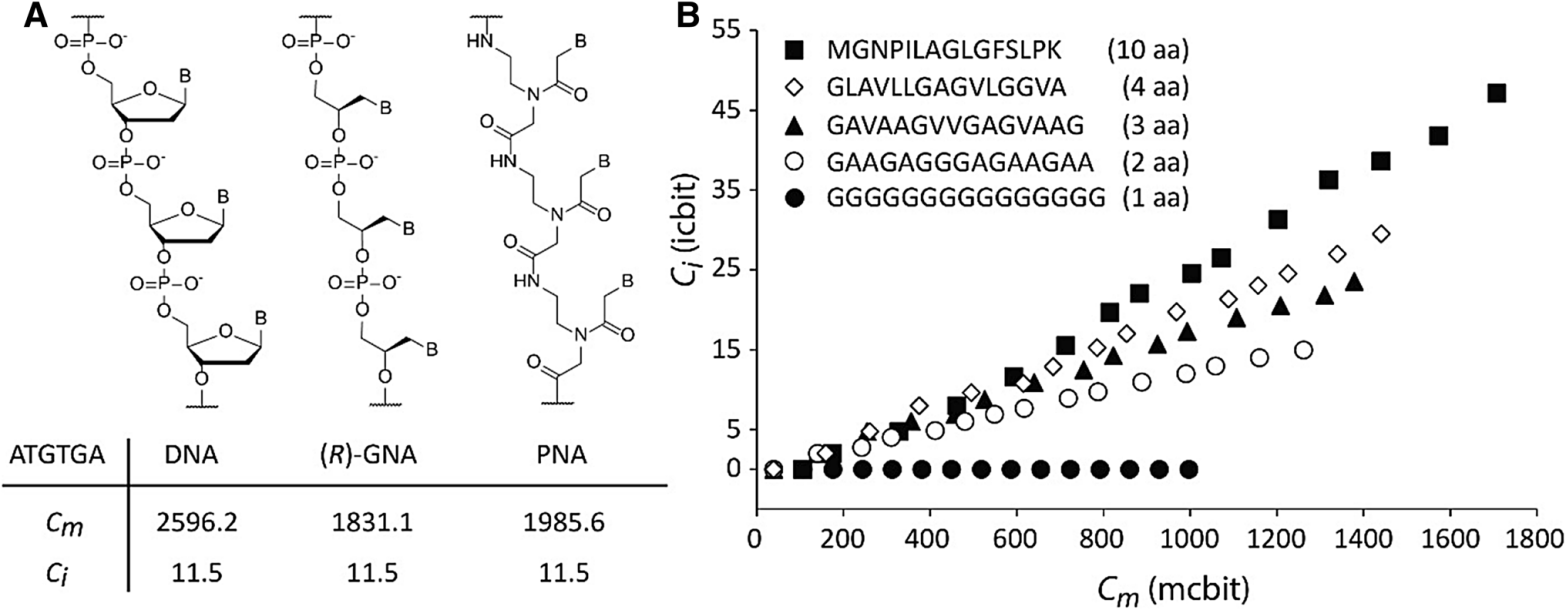
Quantification of molecular (*C*_m_) and information complexity (*C*_*i*_) for various types of biogenic units. **a** Chemical structures of DNA, (*R*)-GNA, and PNA and calculated complexity values for an arbitrary model sequence (ATGTGA). **b** Information complexity plotted against molecular complexity for arbitrary protein sequences of different alphabet sizes (1 aa to 10 aa) as function of length. B: nucleobase, aa: amino acid

The quantification of biogenic units is not only possible for extant organisms but also for artificial or potentially prebiotic information carriers like polyamide nucleic acid (PNA) (Nielsen et al. 1991) or glycol nucleic acid (GNA) (Zhang et al. 2005), demonstrating the universal applicability of this concept (Fig. 2a). Calculations for a short model sequence, for example, resulted in considerably lower molecular complexity for GNA and PNA in comparison to a DNA sequence with the same information complexity. Hereby, the complexity scale also allows universally assessing biogenic units with different sizes of alphabet. For example, a homoglycine sequence with “G” as only letter has no information complexity (*C*_*i*_ = 0), while *C*_*i*_ increases in dependence of sequence length for all non-repetitive sequences. Thereby a peptide with a binary (two letter) code naturally has lower information complexity as compared to peptides comprising a larger amino acid alphabet (Fig. 2b). Peptides and proteins based on a reduced alphabet of amino acids have been discussed in the context of prebiotic chemistry and early evolution (Longo et al. 2013).

**Fig. 3:**
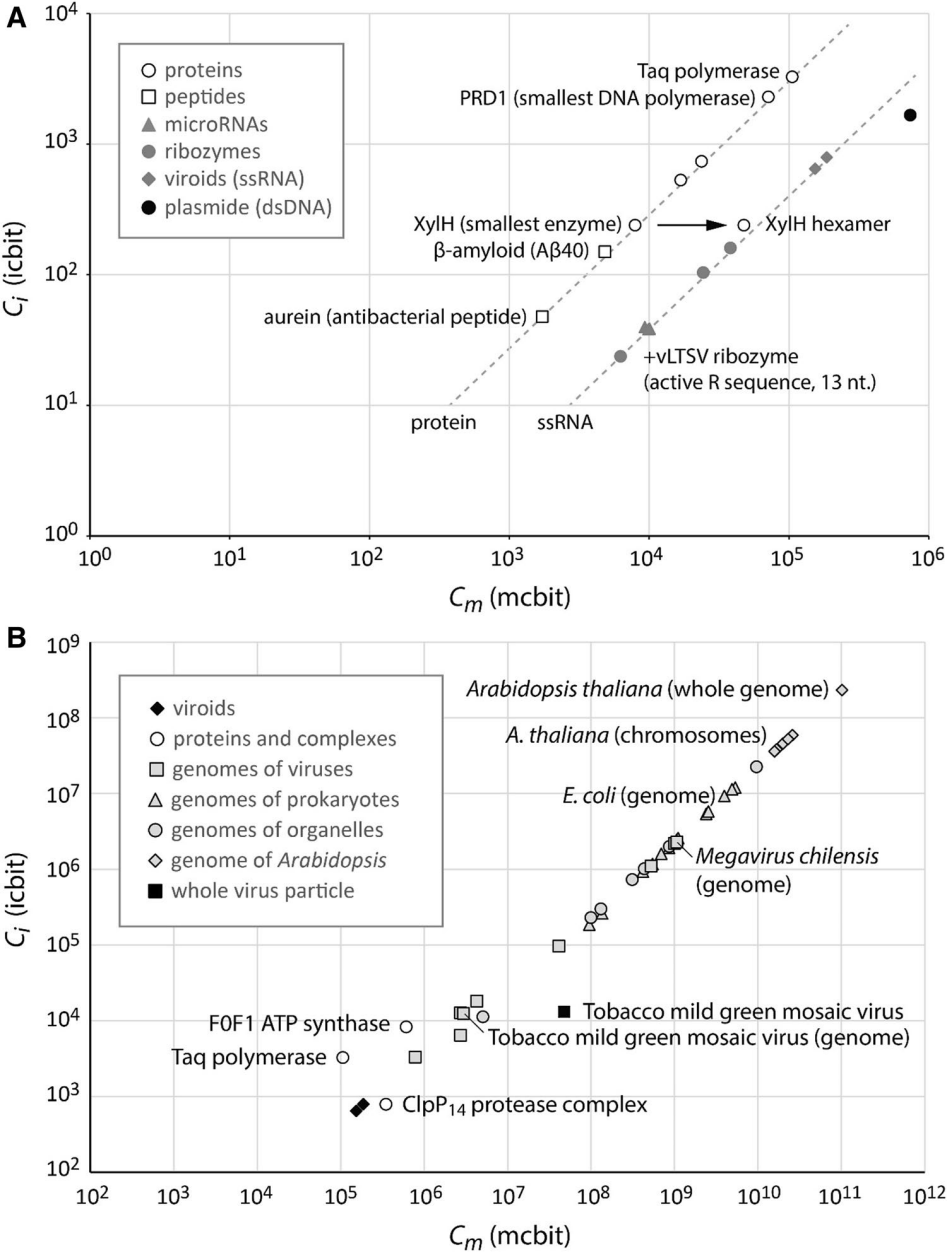
Complexity values calculated for various biogenic units. **a** Lower range of complexity of biogenic units and **b** complexity of larger units such as multimeric proteins, genomes, and an entire virus particle

Applying Eqs. (1) and (3) also allowed calculating the complexities of some of the smallest known biogenic units with biological function (Fig. 3a). Nucleic acids and proteins start with an offset given by the molecular complexity of their monomeric units and both increase with the sequence length of biogenic units. Thus, the value of molecular complexity is always somewhat larger than that of information complexity. The molecular complexity of monomers differs between different biopolymers resulting in discrete compound class-dependent shifts in the complexity space. Double-stranded DNA (dsDNA) can be interpreted as a structure composed of base pairs as largest non-repetitive units and is thus again shifted by a few mcbit of molecular complexity from single-stranded nucleic acids (ssRNA or ssDNA) with the same information complexity.

Small peptides like aurein with a sequence of only 16 amino acid or small RNAs with around 20 nucleotides form the lower rage of complexity of biogenic units. The smallest catalytically active biogenic units are the hammerhead ribozymes and a 13 nucleotide sequence of the + vLTSV ribozyme (Jeffries and Symons 1989). While these ribozymes only cleave the sugar-phosphate backbone of specific RNA sequences, higher levels of complexity seem to be required to enable other catalytic activities. The smallest known protein enzyme XylH with 63 amino acids assembles into the hexameric XylH_6_ complex that catalyzes the isomerization of a metabolite. Formation of complexes containing several identical subunits creates new biogenic units leading to an increase in molecular complexity but not in information complexity. Even the smallest biogenic units that are capable of accurately replicating other biogenic units like the DNA polymerase PRD1 have complexities of more than one order of magnitude larger in both *C*_m_ and *C*_*i*_ than the smallest natural peptides or microRNAs (Fig. 3a).

Like viruses, viroids are cellular parasites but only consist of a single circular RNA molecule without additional proteins and thus can be regarded as individual units of natural selection with one of the lowest complexities. In contrast, viruses have larger genomes encoding various proteins for their capsids and their infectious lifestyle.

Larger multiprotein complexes like the F0F1 ATP synthase composed of 22 subunits with 8 different proteins are already in the complexity range of viral genomes (Fig. 3b). In case of the tobacco mild green mosaic virus, the virus capsid is well defined and consists of 2130 copies of a single type of protein. Thus, even the total complexity of the entire virus particle can be calculated (Fig. 3b).

While the information complexity of the virus particle did not increase strongly in comparison to that of the viral genome, the molecular complexity increased by more than an order of magnitude. Also the complexities of the genomes of prokaryotic and eukaryotic cells as well as of organelles can be calculated (Fig. 3b). The smallest prokaryotic genomes of certain endosymbionts are overlapping with viral and organelle genome complexities and the genome of one of the largest existing viruses, *Megavirus chilensis* marks currently the upper limit known for viruses. The complexity of the genomes of the largest viruses is thus higher than that of the genomes of some cell organelles and prokaryotic species. The complexity further increases with increasing size of the genomes from prokaryotes to eukaryotes (Fig. 3b).

For larger biogenic units like organelles, entire cells, and multicellular organisms, RNA and protein expression levels fluctuate and strongly depend on environmental conditions. These units do not have a defined molecular composition and their molecular complexity is subject to variation. Additionally, for most organisms even the compositional range, such as the numbers of proteins, RNAs, lipids, and carbohydrates per cell, is not precisely known. One also must account for errors and noise in the information content, which is resulting from continuous interactions of the genome with the environment. Thus, cells and organisms experience a large extent of non-linearity, which impedes the reliability of determining exact values for *C*_m_ and *C*_*i*_. However, the complexity of these biogenic units can be roughly estimated within an order of magnitude by using approximations. Sequence information complexity is dominated by genome sequences and was further corrected by adding the information complexity of the encoded proteins. Epigenetic information constitutes an important additional layer of information complexity. Yet, epigenetic information has not been deciphered completely for any organism so far and the epigenetic modifications in a cell are intrinsically unstable and matter of continuous remodeling, and was thus not included into *C*_*i*_. In principle, however, it should be possible to include epigenetic information into calculations of information complexity term.

**Fig. 4:**
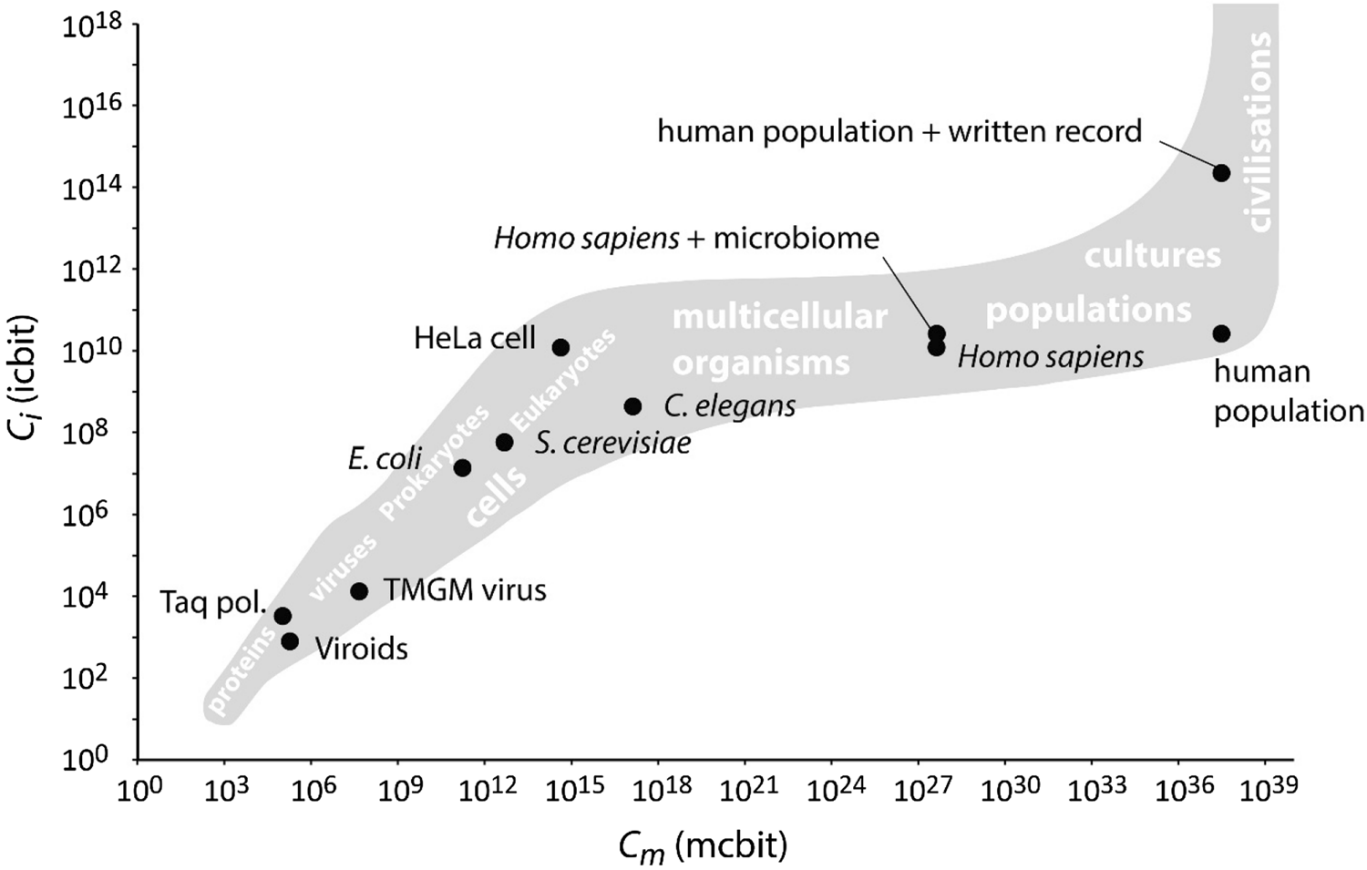
Universal complexity scale plot with representative biogenic units of Earth’s biosphere

In contrast to information complexity, molecular complexity is mainly determined by the cellular protein content. Typically, proteins constitute the largest fraction of a cell’s components with approximately 3 × 10^6^ proteins per µm^3^ of cell volume (Milo 2013). The cell volume and the median size of proteins have been reported for different species and can be used for estimating the molecular complexity of a cell (Brocchieri and Karlin 2005). For these calculations, averaged molecular complexity values of 129.3 mcbit per amino acid and 850.3 mcbit per base pair of dsDNA and information complexity values of 4.02 icbit per amino acid and 1.96 icbit per base pair of dsDNA were applied. Using these approximations, the complexities of various biogenic units on organism level were calculated (Fig. 4). These range from unicellular organisms, such as *E. coli* as representative of prokaryotes, the yeast *Saccharomyces cerevisiae* as eukaryotic member, and the human cancer cell line HeLa to multicellular organisms, such as the nematode *Caenorhabditis elegans* and *Homo sapiens*. From unicellular to multicellular biogenic units, molecular complexity increases over-proportional to information complexity. Although the human body can be seen as a consortium of human cells and microbial cells that form a unit, even maximum estimates demonstrate that due to the relatively simple complexity of bacteria compared to a human cell the overall complexity of a human individual is not considerably affected (Fig. 4). If we consider the human species as a single unit that is defined by globally interacting subunits (humans) and these interactions determine its structure and stage of development, molecular complexity increases further by including the entire human world population. In contrast, prokaryotic populations or groups of social animals may be restricted in their interactions via chemical signals and direct physical interactions to much smaller local scales. Genetic variation within the human species was not taken into account but may lead only to a slight increase in information complexity. Human culture, such as, for example, language may add an additional level of information complexity that is defining an important evolutionary component of our civilization (Komarova 2006). As an example, the information content of the entirely written record published in books was included into the estimations of sequence information complexity (Fig. 4). This measure strongly increased information complexity while it had no effect on molecular complexity. Thus, any biogenic unit can be represented by information complexity and molecular complexity of its biogenic units. The universal complexity scale thereby allows displaying and correlating the whole spectrum of biogenic units ranging from molecules via subunits to organisms and populations including emergent social phenomena like language or technology. The relative placement of a unit in this universal complexity space may convey information about the minimum level of complexity of biogenic units of a biosphere. While it is disputable if there is a general tendency towards the increase of complexity during evolution (Emmeche 1997), it is undoubted that major transitions in evolution, such as the formation of the first cells, the evolution of Eukaryotes, or the rise of multicellular organization forms, have been accompanied with increasing levels of complexity (Szathmary 2015). Any potential biogenic unit in the Universe can now be correlated in the complexity space with the levels calculated for biogenic units on Earth.

## Operational Value

The concept of biogenic units along with quantifying the complexity of these units via orthogonal measures of complexity provides an important operational tool and theoretical framework for the search for life in the Universe and the study of the origins of life. As such, it allows quantifying the complexity of units ranging from individual molecules to molecular assemblies in a gradual manner, without being bound by any specific definition of life. Hereby life is a systems property which manifests in a continuum of units and subunits with different degrees of complexities. The universal complexity scale proposed here will aid our understanding of this gradualism from molecules to life-like systems regarding the origins of life as well as the reticulated network of subunits and units of extant life. It is thus irrelevant if any individual unit or subunit, e.g., a virus particle would be considered life or alive. Increasingly complex biogenic units necessitate sufficiently complex mechanisms allowing their efficient and accurate production. The complexity of such a mechanism may gradually increase with the complexity of the sequence information of a biogenic unit. Multicellular organisms, for instance, require more elaborate mechanisms for their reproduction in comparison to unicellular prokaryotes. Yet, prokaryotes require again more complex processes for their replication as compared to short nucleic acid molecules. Different processes such as cross-catalysis may have been at work at the transitions from prebiotic chemistry to biological evolution (Lincoln and Joyce 2009; Yao et al. 1998) and in an astrobiological context, putative life as we do not know it may potentially involve entirely different mechanisms to generate complex biogenic units. Hereby, the common scale of complexity may help to quantify and compare such units with the units of known life on Earth.

The evolutionary stage of a selected system or even an entire biosphere can thus be estimated by maximum complexity (*C*_m,max_; *C*_*i*,max_) of its biogenic units. If, for instance, the maximum complexity of biogenic units discovered on a planet is below that of functional enzymes, it may indicate at a prebiotic stage of development. In contrast, a much higher level of maximum complexity may indicate, for example, a stage equivalent or comparable to multicellular organisms regardless of the chemistry, molecular mechanisms, and organization forms involved in its production and maintenance. The universal scale of complexity may thus provide a valuable tool for the detection, identification, and quantitative comparison of prebiotic and life-like systems in the Universe.

Any level of complexity builds on the achievements of previous transition events, and thus also includes biogenic units of lower complexity levels.

Malaterre suspected gradualism at the roots of the tree of life and proposed “lifeness signatures” as a simple way of measuring evolutionary milestones from non-living to living matter (Malaterre 2010). The here introduced concept of complexity of biogenic units takes this idea further and allows to directly quantify and correlate units of any prebiotic or life-like system on a common scale.

In addition, this approach also may provide a framework to investigate the transitions from prebiotic chemistry to early life. Even the smallest biogenic units on present-day Earth with significant catalytic activity have a degree of information complexity (Fig. 3a) that is highly unlikely to emerge from statistical combination of their building blocks. Thus, ancestral, simpler units must have existed that gradually led to the evolution of efficient and complex modern biocatalysts.

Hereby, the universal complexity scale may help to discover potential trajectories in complexity space leading from prebiotic units of low complexity to biogenic units with high complexity. While proteins may be close to a limit of maximum information density (*C*_*i*_/*C*_m_), it may be conceivable investigating routes via molecular assemblies starting with lower information density based on greater molecular complexity that ultimately allowed a gradual increase in information complexity. Such a potential solution could be compositional information of non-covalently assembled units like composomes or substrate channeling by units of simple catalytic networks (Edwards 1996; Segre et al. 2000; Segre and Lancet 2000). Compositional information could be integrated with sequence information or alternatively be added as another orthogonal dimension of complexity. A further possible orthogonal dimension may be functional information, which in some cases could provide important insights into evolution (Hazen et al. 2007). Applying multidimensional orthogonal measures on investigating the origins of life could thus provide different perspectives on the gradual functional, compositional, and structural transitions from prebiotic chemistry to life.

## Summary

In conclusion, the concept of biogenic units with orthogonal complexity measurements creates a common scale that allows to compare the complexity values of chemical and biological units. It may thereby provide a powerful framework aiding the detection, identification, and quantitative comparison of prebiotic and life-like systems in the Universe. This approach will inform the search for life in the Universe and may help developing new concepts of the origins of life.

## Methods

Genomic and protein data were retrieved from the National Center for Biotechnology Information (NCBI) public databases http://www.ncbi.nlm.nih.gov. Simple additive terms for molecular complexity of amino acids in a peptide or protein sequence and nucleotides in RNA or DNA are provided (Tables S1 and S2). Further details on complexity calculations and datasets are provided in the Supporting Information (Tables S3–S8).

## Electronic supplementary material

Below is the link to the electronic supplementary material.

Supplementary material 1 (DOCX 446 KB)
