# Supplementary material for: From Molecules to Life: Quantifying the Complexity of Chemical and Biological Systems in the Universe
Source: J Mol Evol. 2017 Dec 19;86(1):1–10. doi: 10.1007/s00239-017-9824-6 (PMC5794832; doi:10.1007/s00239-017-9824-6)
Supplement: Supplementary file 1 — Supplementary material 1 (DOCX 446 KB) [file 239_2017_9824_MOESM1_ESM.docx]

Supporting Information

**Quantifying the complexity of chemical and biological systems in the Universe**

Thomas Böttcher^1,^*

^1^Department of Chemistry, Konstanz Research School Chemical Biology,

Zukunftskolleg, University of Konstanz, Germany

*Corresponding author

E-mail: [thomas.boettcher@uni-konstanz.de](mailto:thomas.boettcher@uni-konstanz.de)

Genomic and protein data were retrieved from the National Center for Biotechnology Information (NCBI) public data base <http://www.ncbi.nlm.nih.gov>.

Molecular complexity of various biomolecules was calculated using equation (Eq. 1). Detailed discussion of molecular complexity and instructions for the application of the equation are provided in a separate paper (Böttcher 2016). Simple additive terms for molecular complexity of amino acids in a peptide or protein sequence and nucleotides in RNA or DNA are given in tables S1 and S2, respectively. The total complexity of a sequence can be simply calculated by adding the corresponding terms and correcting for the terminal residues by the appropriate correction terms (Tables S1 and S2).

For various biogenic units the corresponding values calculated for molecular and information complexity are given below (Tables S3-S6). For complexity calculations of large genomes, the numbers of nucleotides were estimated from the genome size and the GC content. In double stranded DNA information does not increase with the second strand while molecular complexity of a corresponding single strand is doubled. Similarly, information complexity does not change when multimeric subunits of the same type assemble, while molecular complexity increases directly with every subunit that forms in interaction with other subunits a new biogenic unit.

Complexities of entire organisms were estimated using approximations for the numbers and average sizes of the genome, proteins, RNAs, and lipids in a cell. Phosphatidylethanolamine was used as sample lipid for approximating the molecular complexity of an average lipid molecule in the cell membrane. Detailed calculations for *Escherichia coli* (Table S7) demonstrated, that lipids only contributed approx.10% of the total molecular complexity. The genome dominated information complexity and the proteins were the major contributors to molecular complexity. Thus, for further calculations, lipids and RNA could be neglected (Table S8) leading estimated to only minor deviations in the final values much less than an order of magnitude. Epigenetic information is currently not well enough understood to be included in information complexity. It should thus be considered that information complexities of entire organisms are rather underestimated. For Eukaryotic cells a diploid set of chromosomes was used for calculations and as simplification the information content was assumed for the diploid cell. Specialized cell types of higher organisms were not taken into account and also genetic variation that may increase the information complexity in a population was not considered.

**Table S1. Calculated molecular complexity of amino acid residues within a peptide or protein sequence with correction terms for the N-terminal NH_2_ and the C-terminal carboxylate group.**

| Amino acid residue in sequence | Molecular complexity  (mcbit) |
| --- | --- |
| A | 101.477 |
| R | 159.128 |
| N | 153.291 |
| D | 127.817 |
| C | 116.647 |
| Q | 159.291 |
| E | 133.817 |
| G | 68.458 |
| H | 187.308 |
| I | 130.987 |
| L | 120.232 |
| K | 134.121 |
| M | 135.817 |
| F | 131.402 |
| P | 133.631 |
| S | 116.647 |
| T | 149.666 |
| W | 209.784 |
| Y | 148.987 |
| V | 114.232 |
| Correction term N-terminus | -8.644 |
| Correction term C-terminus | -20.830 |

**Table S2. Calculated molecular complexity of nucleotides within a nucleic acid (DNA or RNA) with correction terms.**

| Nucleotide in nucleic acid sequence | | Molecular complexity  (mcbit) |
| --- | --- | --- |
| DNA | dA | 435.778 |
|  | dT | 411.540 |
|  | dG | 462.269 |
|  | dC | 392.523 |
| RNA | A | 477.968 |
|  | U | 446.899 |
|  | G | 504.458 |
|  | C | 434.713 |
|  | PP* addition | 82.288 |
|  | Terminal phosphate correction | -28.137 |
|  | Terminal sugar correction | 5.170 |

*Diphosphate

**Table S3. Molecular complexity and information complexity calculated for various biogenic units.**

| Biogenic unit | Molecular complexity  (mcbit) | Information complexity (icbit) |
| --- | --- | --- |
| **Ribozymes** |  |  |
| Hammerhead AJ247116.1 | 38135 | 160.39 |
| Hammerhead AJ550909.1 | 24393 | 103.78 |
| vLTSV ribozyme | 6245 | 23.74 |
| **Micro RNAs** |  |  |
| hcmv-miR-UL22A-5p | 9310 | 39.71 |
| hsa-miR-1-5p | 10062 | 38.56 |
| mmu-mir-142a | 9879 | 38.26 |
| **Small peptides** |  |  |
| Aurein | 1756 | 47.74 |
| beta-Amyloid | 4872 | 150.08 |
| **Proteins** |  |  |
| Taq Polymerase P19821-1 | 106436 | 3276.08 |
| 2-Hydroxymuconate tautomerase XylH | 7988 | 239.56 |
| Glucagon (*H*. *sapiens*) | 23841 | 737.90 |
| Lysozyme (*H*. *sapiens*) | 16913 | 530.78 |
| PRD1 DNA-Polymerase | 71543 | 2301.64 |
| **Protein complexes** |  |  |
| XylH_6_ (*P. putida*) | 47928 | 239.56 |
| ClpP_14_ (*S*. *aureus*) | 349537 | 790.02 |
| F_0_F_1_ ATP Synthase (*E*. *coli*) | 609676 | 8307.08 |
| **Plasmide** |  |  |
| Thermotoga RQ7 plasmide | 737291 | 1667.42 |
| **Viroids** |  |  |
| Apple scar skin viroid | 153623 | 647.46 |
| Chrysanthemum chlorotic mottle viroid | 186151 | 794.64 |

**Table S4. Molecular complexity and information complexity calculated for multiunit composed biogenic units.**

| Biogenic unit | | Molecular complexity  (mcbit) | Information complexity (icbit) |
| --- | --- | --- | --- |
| **F_0_F_1_ ATP Synthase (*E*. *coli*)** | |  |  |
|  | ATP Synthase subunit a_1_ | 34776 | 1081.71 |
|  | ATP Synthase subunit b_2_ | 39709 | 583.58 |
|  | ATP Synthase subunit c_10_ | 93397 | 282.63 |
|  | ATP Synthase subunit alpha_3_ | 192314 | 2075.78 |
|  | ATP Synthase subunit beta_3_ | 173245 | 1862.50 |
|  | ATP Synthase subunit gamma_1_ | 36458 | 1171.83 |
|  | ATP Synthase subunit delta_1_ | 22267 | 698.06 |
|  | ATP Synthase subunit epsilon_1_ | 17509 | 550.98 |
|  | Total | 609676 | 8307.08 |
| **Tobacco mild green mosaic virus** | |  |  |
|  | Genome (ssRNA) | 2957413 | 12557.30 |
|  | Capside – 2130mer | 44245760 | 624.85 |
|  | Total virus | 47203173 | 13182.15 |

**Table S5. Molecular complexity and information complexity calculated for the genomes of various biogenic units. Molecular complexity is given for dsDNA unless indicated otherwise.**

| Biogenic unit (genomes) | Molecular complexity  (mcbit) | Information complexity (icbit) |
| --- | --- | --- |
| **Bacteria** |  |  |
| *Escherichia coli* | 3948892195 | 9278514.47 |
| *Staphylococcus aureus* | 2397527332 | 5399806.81 |
| *Pseudomonas aeruginosa* | 5339123954 | 12021147.30 |
| *Mycoplasma gallisepticum* | 858168932 | 1917849.65 |
| *Mycoplasma genitalium* | 492883108 | 1102776.21 |
| *Mycoplasma pneumoniae* | 694185928 | 1609072.24 |
| *Chlamydophila pneumoniae* | 1061707422 | 2465077.62 |
| *Carsonella ruddii* | 135481330 | 262825.70 |
| *Nasuia deltocephalinicola* | 95119929 | 186069.63 |
| *Wolbachia* | 1077548032 | 2454221.20 |
| *Buchnera aphidicola* | 545045546 | 1175236.57 |
| **Archaea** |  |  |
| *Methanosarcina acetivorans* | 4891697595 | 11414230.80 |
| *Methanothermus fervidus* | 1056442448 | 2362323.00 |
| *Ignicoccus hospitalis* | 1104906421 | 2579213.15 |
| *Sulfolobus solfataricus* | 2543389157 | 5807978.98 |
| *Nanoarchaeum equitans* | 417094988 | 932670.92 |
| **Organelles** |  |  |
| Ch *Paulinella chromatophora* | 868534268 | 2000294.87 |
| Mt *Capsicum annuum* | 435130576 | 1018618.71 |
| Chl *Pinus koraiensis* | 99636984 | 230102.18 |
| Mt *Silene conica* | 9626402977 | 22480023.50 |
| Mt *Plasmodium falciparum* | 5069997 | 11337.84 |
| Mt *Arabidopsis* | 312129877 | 730980.04 |
| Chl *Arabidopsis* | 131311089 | 300482.15 |
| **Viruses** |  |  |
| Enterobacteria phage lambda | 41277457,9 | 97003.86 |
| Acanthamoeba polyphaga mimivirus | 1003620279 | 2192306.05 |
| Human Hepatitis B virus | 2735927,47 | 6429.55 |
| Megavirus chiliensis | 1069314354 | 2284733.64 |
| CroV (Cafeteria roenbergensis virus BV-PW1) | 524254224 | 1101044.93 |
| Abaca bunchy top virus (ssDNA) | 2730564 | 12694.43 |
| Hepatitis delta virus (ssRNA) | 784946 | 3326.22 |
| Human immunodeficiency virus 1, HIV-1 (ssRNA) | 4273304 | 18195.98 |
| Tobacco mild green mosaic virus (ssRNA) | 2957413 | 12557.30 |

Mt: mitochondria, Ch: chromatophore, Chl: chloroplast

**Table S6. Molecular complexity and information complexity calculated for the haploid genomes of Eukaryotic biogenic units including cell organelles.**

| Biogenic unit (genomes) | Molecular complexity  (mcbit) | Information complexity (icbit) |
| --- | --- | --- |
| ***Arabidopsis thaliana*** |  |  |
| Chromosome 1 | 2.5866•10^10^ | 59090495 |
| Chromosome 2 | 1.6745•10^10^ | 38254445 |
| Chromosome 3 | 1.9942•10^10^ | 45633108 |
| Chromosome 4 | 1.5802•10^10^ | 36145110 |
| Chromosome 5 | 2.2933•10^10^ | 52391113 |
| Mitochondrion | 312129877 | 730980 |
| Chloroplast | 131311089 | 300482 |
| Total genome | 1.0173•10^11^ | 232545732 |
| ***Saccharomyces cerevisiae* S288c** |  |  |
| Chromosome 1 | 195744057 | 452772 |
| Chromosome 2 | 691353166 | 1593949 |
| Chromosome 3 | 269188877 | 621049 |
| Chromosome 4 | 1300730398 | 2994719 |
| Chromosome 5 | 490455639 | 1131537 |
| Chromosome 6 | 229693686 | 530282 |
| Chromosome 7 | 926680433 | 2135032 |
| Chromosome 8 | 478356508 | 1103623 |
| Chromosome 9 | 374003918 | 864007 |
| Chromosome 10 | 634028519 | 1462282 |
| Chromosome 11 | 566903981 | 1306123 |
| Chromosome 12 | 918211065 | 2118417 |
| Chromosome 13 | 785926285 | 1811369 |
| Chromosome 14 | 666842195 | 1538994 |
| Chromosome 15 | 926688580 | 2135792 |
| Chromosome 16 | 806013038 | 1857020 |
| Mitochondrion | 72791717 | 142392 |
| Total genome | 1.0334•10^10^ | 23799358 |

**Table S7. Estimations for molecular complexity (mcbit) and information complexity (icbit) for an *E. coli* K12 cell.**

|  | Genome | Proteins | tRNA | rRNA | Lipids |
| --- | --- | --- | --- | --- | --- |
| different molecules | 1 | 4140 | 89 | 22 | - |
| average size/molecule | 4639.7 kbp | 278 aa | 80 nt | 4520 nt | - |
| mcbit/molecule | 3.95•10^9^ | 35954 | 37296 | 2107204* | 434 |
| icbit/molecule | 9.28•10^6^ | 1117 | 158 | 8945* | 0 |
| molecules per cell | 1(dsDNA) | 3•10^6^ | 2•10^5^ | 2•10^4^ | 3•10^7^ |
| mcbit/cell | 3.95•10^9^ | 1.08•10^11^ | 7.46•10^9^ | 4.21•10^10^ | 1.30•10^10^ |
| icbit/cell | 9.28•10^6^ | 4.62•10^6^ | 14090 | 8945 | 0 |

*per ribosome, kbp: kilobase pairs, nt: nucleotides, aa: amino acids

**Table S8. Approximations and estimations for molecular complexity (mcbit) and information complexity (icbit) for Eukaryotic cells.**

|  | Genome | Proteins |
| --- | --- | --- |
| ***Homo sapiens* HeLa cell** | |  |
| different molecules | 2* | 25000 |
| average size/molecule | 3.03•10^9^ bp | 375 aa |
| mcbit/molecule | 2.58•10^12^ | 48499 |
| icbit/molecule | 0.59•10^10^ | 1506 |
| molecules per cell | 2* | 9•10^9^ |
| mcbit/cell | 5.16•10^12^ | 4.36•10^14^ |
| icbit/cell | 1.19•10^10^ | 3.77•10^7^ |
| human body: ~10^13^ cells | | |
| human microbiome: ~10^13^ cells, 1000 species | | |
| human world population: ~7•10^9^ | |  |
| ***Saccharomyces cerevisiae*** | |  |
| different molecules | 2* | 5907 |
| average size/molecule | 1.25•10^7^ bp | 379 aa |
| mcbit/molecule | 1.06•10^10^ | 49016 |
| icbit/molecule | 2.45•10^7^ | 1522 |
| molecules per cell | 2* | 1•10^8^ |
| mcbit/cell | 2.13•10^10^ | 4.90•10^12^ |
| icbit/cell | 4.90•10^7^ | 8.99•10^6^ |
| ***Caenorhabditis elegans*** | |  |
| different molecules | 2* | 27368 |
| average size/molecule | 1.00•10^8^ bp | 344 aa |
| mcbit/molecule | 0.85•10^11^ | 44490 |
| icbit/molecule | 1.97•10^8^ | 1382 |
| molecules per cell | 2* | 3•10^9^ |
| mcbit/cell | 1.71•10^11^ | 1.33•10^14^ |
| icbit/cell | 3.93•10^8^ | 3.78•10^7^ |
| worm: 1031 cells |  |  |

bp: base pairs, aa: amino acids, *diploid genomes, partitioned in chromosomes, dsDNA of each haploid chromosome set is referred to as 1 molecule

**
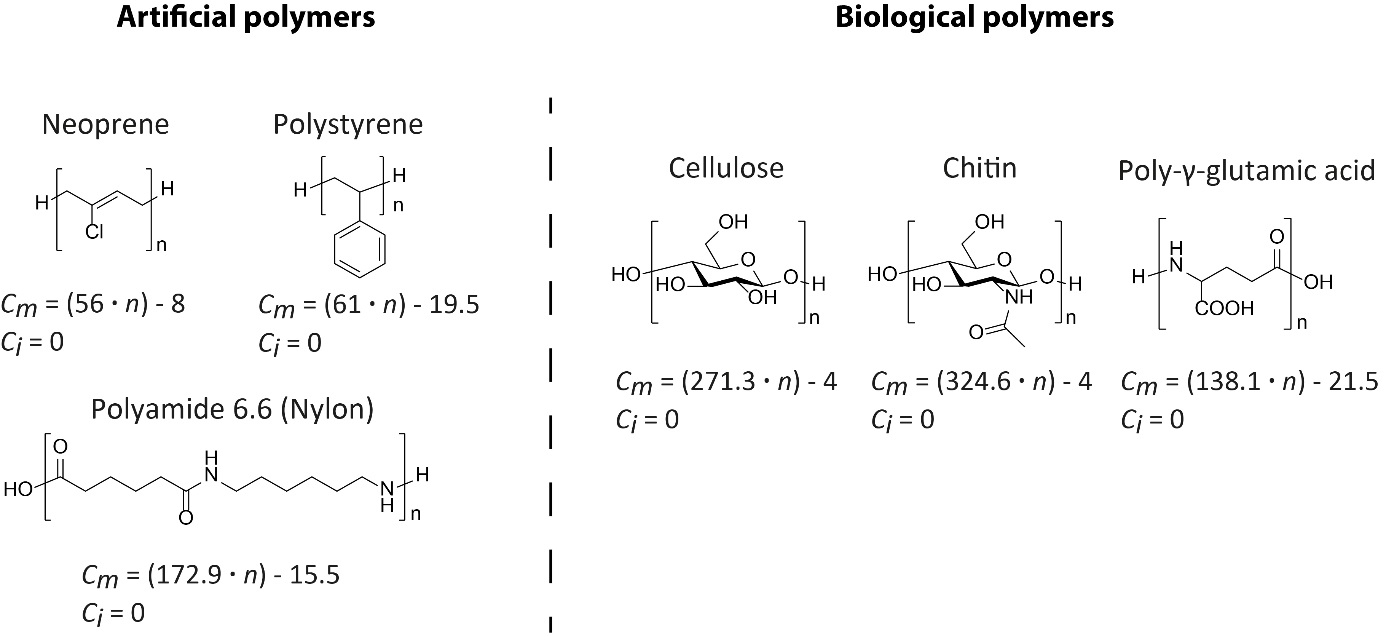
**

**Figure S1:** Molecular complexity for various artificial and biological polymers without sequence information.


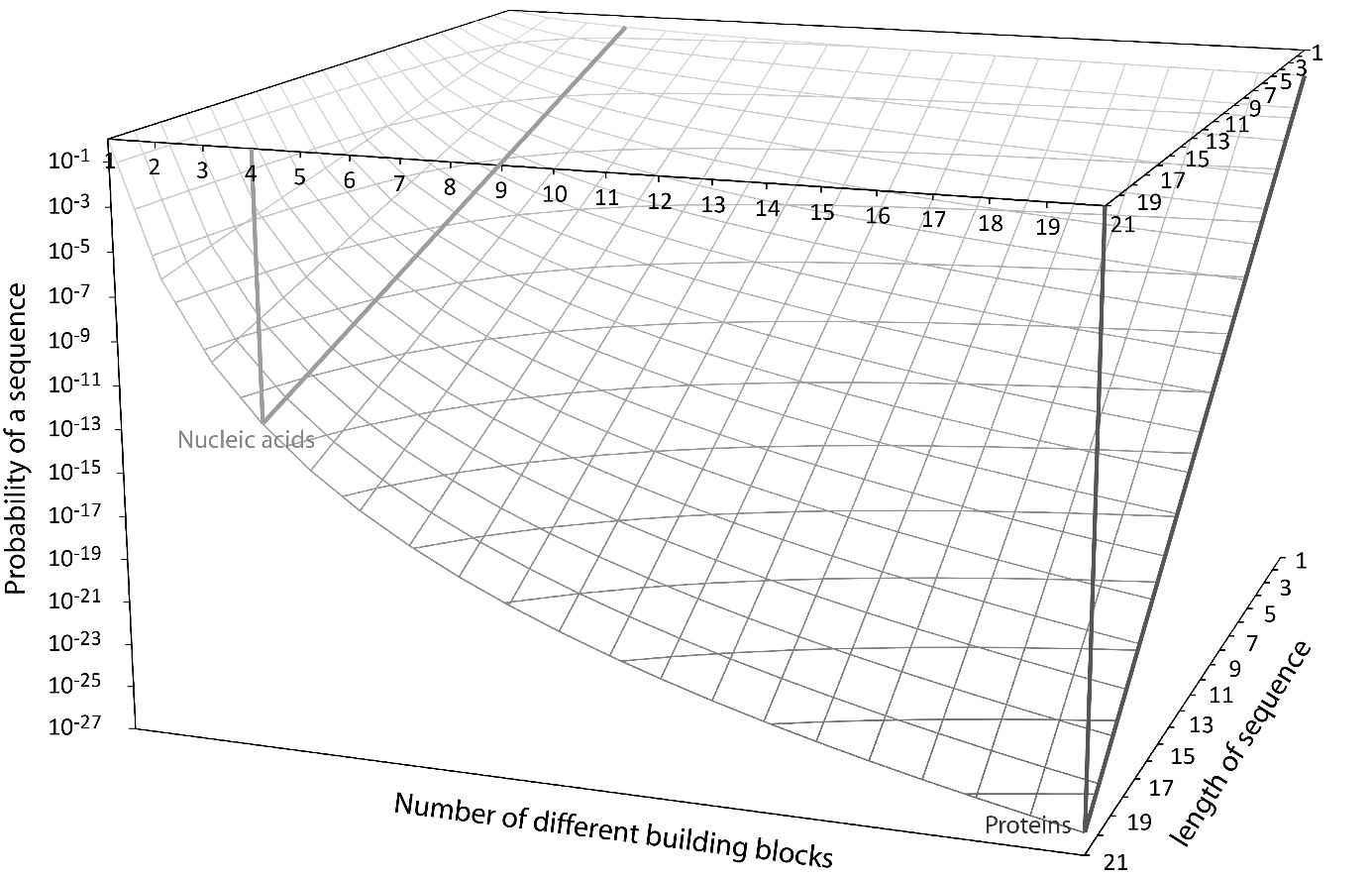


**Figure S2.** Probability of obtaining a defined sequence by combinatorial events without a sequence specific replication or production process in dependence of the number of building blocks (e.g. 4 nucleotides or 20 amino acids) and the length of a sequence.

Reference

Böttcher T (2016) An Additive Definition of Molecular Complexity Journal of chemical information and modeling 56:462-470
